# From Experience to Memory: On the Robustness of the Peak-and-End-Rule for Complex, Heterogeneous Experiences

**DOI:** 10.3389/fpsyg.2019.01705

**Published:** 2019-07-24

**Authors:** Wim Strijbosch, Ondrej Mitas, Marnix van Gisbergen, Miruna Doicaru, John Gelissen, Marcel Bastiaansen

**Affiliations:** ^1^Academy for Leisure, Breda University of Applied Sciences, Breda, Netherlands; ^2^Academy for Tourism, Breda University of Applied Sciences, Breda, Netherlands; ^3^Academy for Digital Entertainment, Breda University of Applied Sciences, Breda, Netherlands; ^4^Tilburg School of Social and Behavioral Sciences, Tilburg University, Tilburg, Netherlands

**Keywords:** experience, memory, experiencing self, remembering self, peak-and-end-rule

## Abstract

Memory forms the input for future behavior. Therefore, how individuals *remember* a certain experience may be just as important as the experience itself. The peak-and-end-rule (PE-rule) postulates that remembered experiences are best predicted by the peak emotional valence and the emotional valence at the end of an experience in the here and now. The PE-rule, however, has mostly been assessed in experimental paradigms that induce relatively simple, one-dimensional experiences (e.g., experienced pain in a clinical setting). This hampers generalizations of the PE-rule to the experiences in everyday life. This paper evaluates the generalizability of the PE-rule to more complex and heterogeneous experiences by examining the PE-rule in a virtual reality (VR) experience, as VR combines improved ecological validity with rigorous experimental control. Findings indicate that for more complex and heterogeneous experiences, peak and end emotional valence are inferior to other measures (such as averaged valence and arousal ratings over the entire experiential episode) in predicting remembered experience. These findings suggest that the PE-rule cannot be generalized to ecologically more valid experiential episodes.

## Introduction

According to Kahneman and Riis (2005), the only thing that we keep from our experiences are the *memories* of those experiences. It is therefore not surprising that the relationship between experience and memory has frequently been addressed in the psychological literature (e.g., see Talarico and Rubin, 2017 for a review on Flashbulb memories and experience). A major finding has been that emotions play an important role in shaping the memory of an experience. Compared to non-emotional experiences, experiential episodes that include emotions are remembered more vividly and in more detail (Kensinger, 2004, 2009; Kensinger and Schacter, 2008). Furthermore, the emotions that are experienced during a certain episode are crucial in determining whether an individual will either repeat or avoid such similar activities and events in the future (Kahneman et al., 1997). Therefore, how individuals *remember* the emotions from their experiences may be just as important as the experienced emotions themselves in guiding subsequent behavior.

What then is the relationship between immediate experience and how experiences are remembered, and which role do emotions play in this relationship? One influential account of this relationship is the *peak-and-end-rule* (Fredrickson and Kahneman, 1993; Fredrickson, 2000), which postulates that how we remember our experiences is determined by the emotions associated with the most intense moment of an experience [the peak] and the emotions associated with the end of an experience [the end]. The *peak-and-end-rule* (henceforth: PE-rule) has been evaluated in many studies and has added substantial insights to the literature. Yet, as we argue, most of these studies have employed very specific experimental paradigms that induce homogeneous and quite unidimensional experiences. This limitation of existing studies questions the generalizability of the PE-rule to the more complex and heterogeneous experiences that make up everyday life. In the present work, we address this issue by assessing the predictive power of the PE-rule in a virtual reality (VR) experience, since VR has been proposed as an excellent way to combine acceptable levels of ecological validity with rigorous experimental control in lab environments (Diemer et al., 2015; Parsons, 2015).

### The Peak-and-End-Rule

The PE-rule was initially proposed by Fredrickson and Kahneman (1993), see also Fredrickson (2000). Earlier studies already suggested that the end of an experience could be relatively more important for how it would be remembered as compared to the entire duration of the experience (Fredrickson, 1990; Hsee and Abelson, 1991; Varey and Kahneman, 1992; Hsee et al., 1994). It was only in 1993, however, that the relative importance of both peaks and ends for memories of experience was explicitly tested. Fredrickson and Kahneman (1993) had participants watch short, plotless movie clips that were either negatively or positively valenced. While watching the movie clips, participants provided an ongoing rating of their affective experience by continuously indicating their emotional valence (the extent to which people feel emotionally positive or negative) through a slider on a response device. Afterward, they provided a rating through self-report for how they remembered their overall emotional valence for the movie clips as a whole. Although Fredrickson and Kahneman (1993) noted that the results would not necessarily exhaust the factors that govern remembered overall emotional valence, it was found that the remembered emotional valence was predicted best by taking the most extreme emotional valence rating [peak] and the emotional valence experienced during the final moments of the experience [end], as well as computing a weighted average of both peak and end.

Much effort has been subsequently devoted to replicating and extending those initial findings. Several follow-up studies used the same approach of providing a continuous rating of experienced emotional valence on the one hand and remembered overall valence on the other hand. Paradigms included watching advertising videos, undergoing colonoscopy, listening to classical/pop music, and hearing annoying sounds (Redelmeier and Kahneman, 1996; Baumgartner et al., 1997; Schreiber and Kahneman, 2000; Rozin et al., 2004). Other studies were conducted in clinical settings, and involved experiences such as bone marrow transplants and giving birth (Ariely, 1998; Chajut et al., 2014). These studies measured ongoing emotional valence through an experience sampling approach (see Hektner et al. (2007) for an overview). Results supported the predictive value of either peaks (Redelmeier and Kahneman, 1996; Baumgartner et al., 1997; Rozin et al., 2004; Chajut et al., 2014), ends (Redelmeier and Kahneman, 1996; Baumgartner et al., 1997; Ariely and Carmon, 2000; Rozin et al., 2004; Chajut et al., 2014), or the average of peaks and ends (Chajut et al., 2014).

Another strand of follow-up studies did not include ratings of ongoing emotional valence, but only included remembered overall valence, or measures such as choice behavior (Kahneman et al., 1993; Redelmeier et al., 2003). In these studies, ongoing emotional valence was assumed to be determined by stimulus parameters, such as holding one’s finger in a closing vise (i.e., yielding increasing negative emotional valence) (Ariely, 1998). As such, changes in stimuli were taken as a proxy for ongoing emotional valence. In these studies, endings in particular have been found to significantly predict remembered overall valence and subsequent behavior (Ariely, 1998; Ariely and Zauberman, 2000), such as whether participants would choose to repeat one experience over another or not (Kahneman et al., 1993). Furthermore, it was found that adding a less painful episode to the end of an already painful procedure (such as holding one’s hand in ice water or undergoing a colonoscopy) would make memories of these painful procedures less negative (Kahneman et al., 1993; Redelmeier et al., 2003). Ariely and colleagues (Ariely, 1998; Ariely and Zauberman, 2000) found changes in final moments of painful and annoying experiences to be significant predictors of remembered evaluations.

In sum, the available evidence supports the notion that peaks and endings are crucial in shaping overall memories of emotional valence. In reviewing early PE-studies, Fredrickson (2000) concludes that the PE-rule is a robust predictor for how experiences are remembered emotionally, as well as for subsequent choice behavior. In addition, the PE-rule offers substantial insights in the context of clinical experiences that may help to mitigate memories of painful experiences, as found by the colonoscopy studies of Redelmeier et al. (2003). As such, the PE-rule has been found to be an established heuristic in closing the gap between the emotions in immediate and remembered experiences.

### Challenges for the Peak-and-End-Rule

More recent studies have challenged the robustness of the PE-rule in various ways (Stone et al., 2000; Cojuharenco and Ryvkin, 2008; Kemp et al., 2008; Seta et al., 2008; Liersch and McKenzie, 2009; Miron-Shatz, 2009; Schneider et al., 2011; Geng et al., 2013). The existing criticism and limitations of the PE-rule can be broadly divided into three different categories. First, the experimental paradigms used to induce experiences have been quite simple, and as a result the induced experiences lack heterogeneity. Second, existing studies have mainly been seeking to confirm the PE-rule and have not systematically included other potential predictors of remembered emotional valence. Third, there is a limited understanding of the robustness of the PE-rule over time. Below, we address each of these criticisms in some detail.

#### Lack of Heterogeneous Experiences as the Object of Study

As noted earlier (Fredrickson, 2000), many studies assessing the PE-rule have used rather short, monotonous experiences in a laboratory setting (Fredrickson and Kahneman, 1993; Baumgartner et al., 1997; Branigan et al., 1997; Ariely, 1998; Ariely and Zauberman, 2000; Rozin et al., 2004). Other studies focused on the experience of physical pain (Kahneman et al., 1993; Redelmeier and Kahneman, 1996; Ariely, 1998; Ariely and Carmon, 2000; Ariely and Zauberman, 2000; Schreiber and Kahneman, 2000; Stone et al., 2000, 2005; Redelmeier et al., 2003; Schneider et al., 2011). As a consequence, Fredrickson (2000) notes that “(…) despite this strong evidence [for the PE-rule], it bears underscoring that these supportive data come from episodes that share specifiable features” (p. 588). With some exceptions, such as listening to music and watching advertisement videos (Baumgartner et al., 1997; Rozin et al., 2004), it can be argued that these specifiable features are too unidimensional and too homogeneous in nature to be representative of the rich diversity of the experiences that make up our everyday life. Generalizations from the early PE-contexts to experiences that lie closer to those we encounter in our everyday lives should therefore be made with caution.

A suggestion that such generalizations may be problematic is offered by Cojuharenco and Ryvkin (2008). They noted in a meta-analysis that peak, end, peak-end, and average scores of immediate emotional valence are often highly correlated. In addition, these correlations become stronger as experiences become more *heterogeneous* in nature. According to Bastiaansen et al. (2019), experiences can be defined as a fine-grained temporal succession of emotions that occur during episodes that we abstract from our human consciousness. The heterogeneity of an experience, then, refers to the richness of an experience, in that it may involve both positively and negatively valenced emotions, and that it may involve qualitatively different emotions (such as anger, fear or joy). Holding one’s finger on a hot stove is a less heterogeneous experience as compared to watching a movie, for instance, as movies generally allow for a multitude of positive and negative emotions. Heterogeneity also means that experiences can differ both between individuals and within individuals over time (Cojuharenco and Ryvkin, 2008). This depends on characteristics of individuals (e.g., personality, mood, attitude), as well as on characteristics of the stimulus configurations.

Arguably, our experiences in everyday life are more heterogeneous in nature compared to the rather simple experiences that have been studied in the PE-context and this may limit the generalizability of the PE-rule. This suggestion is supported by a study employing an ecologically highly valid setting (students going on a 1 week vacation) (Kemp et al., 2008), but also in other contexts where experience is measured over entire days, which involve high heterogeneity (Miron-Shatz, 2009).

#### The Use of Predictors Other Than Peak and End Valence

As said, most existing studies have sought to confirm the PE-rule and as such have only focused on the extent to which emotional peak and end valence predict remembered emotional valence. More recent studies have addressed the predictive value of other measures of immediate experience besides peaks and ends only (Baumgartner et al., 1997; Ariely, 1998; Ariely and Carmon, 2000; Ariely and Zauberman, 2000; Stone et al., 2000, 2005; Rozin et al., 2004; Cojuharenco and Ryvkin, 2008; Kemp et al., 2008; Seta et al., 2008; Miron-Shatz, 2009; Schneider et al., 2011; Hargreaves and Stych, 2013; Chajut et al., 2014). Like peak and end emotional valence, these measures are typically extracted from ratings provided during the experience: the average of all emotional valence ratings [average valence], the variability of the ratings [valence variability], the slope of ratings over time [valence slope], the time it takes from the beginning of an experience to its peak rating [valence peak latency], and the time it takes from its peak rating to the end of the experience [valence end-after-peak latency]. Where most of the studies do indeed find peak, end and average of peak-end to be significant predictors of remembered overall valence, a handful of studies suggests that average valence could be a better predictor for remembered overall valence (Seta et al., 2008; Miron-Shatz, 2009; Schneider et al., 2011). Furthermore, as suggested by Cojuharenco and Ryvkin (2008) from a meta-analysis on the PE-rule, average valence tends to correlate quite highly with peak, end and the average of peak-end.

In addition, a number of potential candidate measures for predicting remembered experience has been overlooked in the literature. First, assuming that the majority of everyday life experiences is likely to be heterogeneous in nature and may include both positive and negative emotions, one should consider not only the peaks in immediate emotional valence, but also the troughs. Second, given its history from the framework of utility studies (for an explanation on the concept of utility, please refer to Kahneman, 2000), studies on the PE-rule have only included the dimension of emotional valence to operationalize experienced affect. This operationalization of affective experience links back to the dimensional approach to emotions (Barrett and Russell, 1999). Originally, however, this approach not only defines emotions in terms of emotional valence but also in terms of emotional activation or *arousal* (Barrett and Russell, 1999). One can easily see that in certain settings, such as riding a roller coaster, white water rafting or watching a horror movie, emotional arousal would potentially have at least as much predictive value in overall evaluations of the experience as emotional valence. Furthermore, it has been shown in the psychological literature that emotional valence enhances memory in a different way and via different brain structures than emotional arousal (Kensinger, 2004, 2009). In sum, in further establishing the robustness of the PE-rule, we will therefore include measures of both emotional arousal as well as emotional valence and we will include measures of positive and negative valence (both peaks *and* troughs, rather than peaks or troughs only).

#### Robustness of the PE-Rule Over Time

A third shortcoming of the PE-rule that limits its generalization to experiences in our everyday life, is that its robustness over time is only marginally studied. Many studies on the PE-rule have only studied remembered overall emotions *directly after the experience*, or at a single point in time only. In daily life, evaluations and decisions are often made long after the initial experience. An example would be that one evaluates one’s summer holiday not only as one gets home, but also at later moments in time, when discussing the holidays with friends and relatives, or even 6 months later, when booking the next vacation. To the best of our knowledge, only one study addressed the robustness of the PE-rule over various moments in time, in the context of vacations (Geng et al., 2013). They found that peaks, ends and peak-ends could only predict remembered overall valence on the short and medium term (the day after the experience had ended, and 3 weeks after the experience had ended) but not on the longer term (7 weeks after the experience had ended). Given the importance of memory at later moments in time for experiences that are ecologically more valid, the lack of empirical data on the robustness of the PE-rule over time is a substantial gap in present knowledge that limits the generalization of this rule to experiences that lie closer to those of everyday life.

### The Present Study

We argue that the literature on the PE-rule is subject to three shortcomings that limit the current understanding of how the PE-rule generalizes to experiences in everyday life. The present work addresses these shortcomings in a study that combines enhanced ecological validity of the induced experience with the rigorous experimental control of a lab environment (Diemer et al., 2015; Parsons, 2015), by using VR to induce an experience. As VR is a medium that leads to high presence in the virtual environment it creates, emotions as experienced in the virtual environment may be closer to emotions experienced in real life situations as compared to traditional media for stimulus presentations. Through a VR device, participants watched a short (14 min) VR movie, and were subsequently asked to retell the movie. Based on their reconstruction, the movie was segmented into episodes, and for each episode measures of emotional valence and arousal were obtained. We then used peaks, troughs, ends, averages and other values for valence and arousal ratings to predict the overall emotional valence and arousal ratings, both immediately after the end of the VR movie and 1 week later. Our study addresses all three shortcomings previously outlined. First, it induces a heterogeneous experience – a virtual reality movie that allows for both positive and negative emotions due to a clearly defined movie plot. Second, it includes all of the predictors that have previously been identified in studies to assess the PE-rule, rather than peaks, ends and the average of peaks and ends only. Furthermore, to have a more complete dimensional approach to affective experience, we include measures of emotional arousal in addition to the commonly used measures of emotional valence. This makes the present work the first PE-study to include emotional arousal besides emotional valence. Third, our study not only includes just one moment for assessing remembered emotional valence and arousal, but an additional second moment 1 week later to assess the robustness of the PE-rule over time. As such, our study puts the PE-rule to a broader empirical test, in an attempt to evaluate its robustness to the listed challenges.

## Materials and Methods

### Participants

Participants consisted of 40 first-year students from Tilburg University, who received study credits for their participation within the study. The group consisted of 16 males and 24 females with age ranging from 19 to 30 (*M* = 21.7; *SD* = 2.8). All participants were fluent in English and had normal or corrected-to-normal vision and hearing. They gave their written informed consent in line with the declaration of Helsinki. As the study included two versions of the VR movie, participants were randomly assigned to one of two groups. One group (*n* = 20) watched the standard version of the movie (regular-version group) and another group (*n* = 20) watched a slightly different version of the movie, in which the key scene was slightly extended in time and intensified in terms of key actions (extended-version group) (see description of the movie below). This manipulation was implemented to see whether suspending and intensifying the main actions in the key scene would elicit stronger emotions and hence stronger peaks and troughs in reported emotional valence and arousal.

### Stimulus Materials

The stimulus materials consisted of two relatively similar versions of the same VR movie entitled *XSTNCE* (Zuijdgeest et al., 2016), which has distinct positively valenced as well as distinct negatively valenced scenes. In the movie, a male and a female scientist are working on the XSTNCE project, which focuses on digitalizing human consciousness so that it can be uploaded into android bodies. In the movie, they succeed in waking their first working android with human consciousness, a role which is fulfilled by the viewers of the movie. The scientists approach the viewer and start to communicate enthusiastically with him/her, realizing that they have just created a major breakthrough [positively valenced part of the movie]. After a while, it appears that the male scientist has a hidden agenda. He wants to delete the consciousness of the android so that he can use it to upload the consciousness of his recently deceased mother, in order to bring her back to life. When the female scientist wants to warn the police, the male scientist kills her by knocking her down [negatively valenced part of the movie]. He then approaches the viewer in order to delete (i.e., kill) its consciousness. As he pulls out a plug, the movie ends with a black screen, followed by the title and credits.

The two stimulus materials differ in terms of the key scene in which the male scientist knocks the female scientist down. In the regular movie (which lasts 13 min and 14 s) the male scientist knocks the female scientist down and she passes out. In the extended movie (which lasts 14 min and 26 s), the male scientist knocks her down, after which she screams for help. The male scientist then repeatedly hits her until she stops moving. For the remainder of the two movies, the scenes from the two different versions are identical to each other. Half of the participants saw the regular version of the movie and the other half saw the extended version. Valence and arousal ratings for both predictor and outcome variables did not differ between the two versions of the movie (see Table 1). As the participants in the two groups also did not differ in terms of background variables (age (*t*_38_ = 0.448; *p* = 0.657) and gender (*t*_38_ = −0.192; *p* = 0.849)), we concluded that this manipulation was not effective and we decided to collapse all data across the two versions of the movie for further data analysis.

**TABLE 1:** Comparison of measures based on valence and arousal ratings between regular-version and extended-version groups.

**Predictor**	**Descriptives for valence**		**Outcomes *t*-tests for valence**	
	***M*(*SD*)_regular_**	***M*(*SD*)_extended_**	***t*_38_**	***p***
Immediate Overall Valence	6.050 (1.356)	5.700 (1.490)	0.777	0.442
Later Overall Valence	5.400 (2.037)	4.750 (2.124)	0.988	0.330
Peak	7.400 (1.046)	7.200 (1.056)	0.922	0.362
Trough	2.400 (1.142)	2.400 (1.095)	0.276	0.784
End	3.450 (1.731)	4.100 (1.943)	−0.952	0.347
Peak-end	5.425 (1.016)	5.650 (1.226)	−0.358	0.722
Trough-end	2.925 (1.248)	3.250 (1.303)	−0.571	0.581
Average	4.961 (0.690)	4.916 (0.947)	0.566	0.574
Variance	3.379 (2.640)	3.277 (2.466)	0.061	0.952
Slope	−0.004 (0.002)	−0.003 (0.002)	−0.855	0.398

**Predictor**	**Descriptives for arousal**		**Outcomes *t*-tests for arousal**	
	***M*(*SD*)_regular_**	***M*(*SD*)_extended_**	***t*_38_**	***p***

Immediate overall arousal	4.400 (2.202)	4.300 (2.227)	0.149	0.882
Later Overall Arousal	5.900 (1.619)	5.450 (1.820)	0.826	0.414
Peak	7.300 (1.261)	6.450 (1.877)	1.777	0.084
End	5.450 (2.235)	4.700 (2.080)	1.032	0.309
Peak-end	6.375 (1.621)	5.575 (1.859)	1.457	0.155
Average	5.370 (1.222)	4.694 (1.741)	1.564	0.126
Variance	2.395 (1.726)	1.832 (1.149)	1.516	0.138
Slope	0.002 (0.003)	0.002 (0.002)	−0.219	0.828

### Design and Procedure

Participants were invited to the lab and were informed that they would watch a movie through a Samsung Gear VR. Having given their informed consent, they were familiarized with the lab environment and the VR device. They were then set up with the VR equipment and were seated in a sound attenuating room so as to be isolated from outside distractions. An experimenter started the movie and left the room. During the watching of the VR movie, contact between participant and experimenter was maintained through a camera and, if necessary, an intercom. At the end of the movie, the experimenter entered the room and took off the VR device.

**FIGURE 1:**
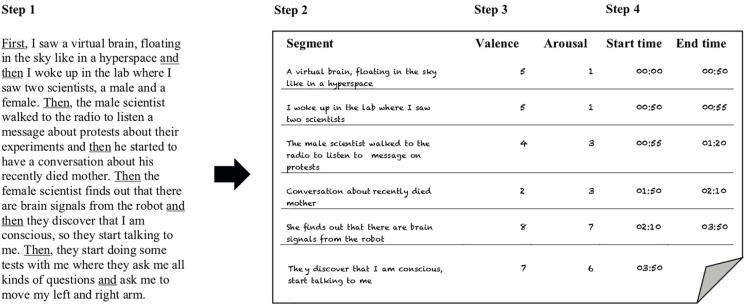
An overview of the segmentation process. Step 1: participants retell their movie experience. Step 2: the experimenter divides this retelling into segments, based on conjunction words used by participants (underlined in Step 1). Step 3: the experimenter asks participants to provide a rating for both emotional valence and arousal, and to base the rating on how they felt while watching that segment. Step 4: both experimenter and participant rewatch the movie, with participants asked to indicate the beginning and ending times of the individual segments.

Directly afterward, participants were asked to retell what they had just experienced from the beginning of the movie to the end and were encouraged to be as detailed as possible. The experimenter simultaneously copied the answers on an answer sheet and was trained to pay attention to conjunction words such as “and then”, “thereafter”, “next,” etc. (see Figure 1). Every time the participant used a conjunction word, the experimenter started to write on a new line. In this way, the story of the participant was divided into segments each and every time a conjunction word occurred. This approach, rather than *a priori* segmenting the movie, takes into account that the experience of the movie might be different for each participant.

In order to extract measures for ongoing experience from participants’ retelling, we used a method inspired by the Day Reconstruction Method (e.g., Kahneman et al., 2004). After participants had finished the retelling, they were first asked to provide an overall evaluation of how they had felt while watching the movie by rating their overall emotional valence and arousal on a 9-point scale, using the assessment procedure described by Bradley and Lang (1994) (*for valence*: 1 = negative, 5 = neutral, 9 = positive, *for arousal*: 1 = calm, 9 = aroused). These measures served as the overall evaluations from memory.

**FIGURE 2:**
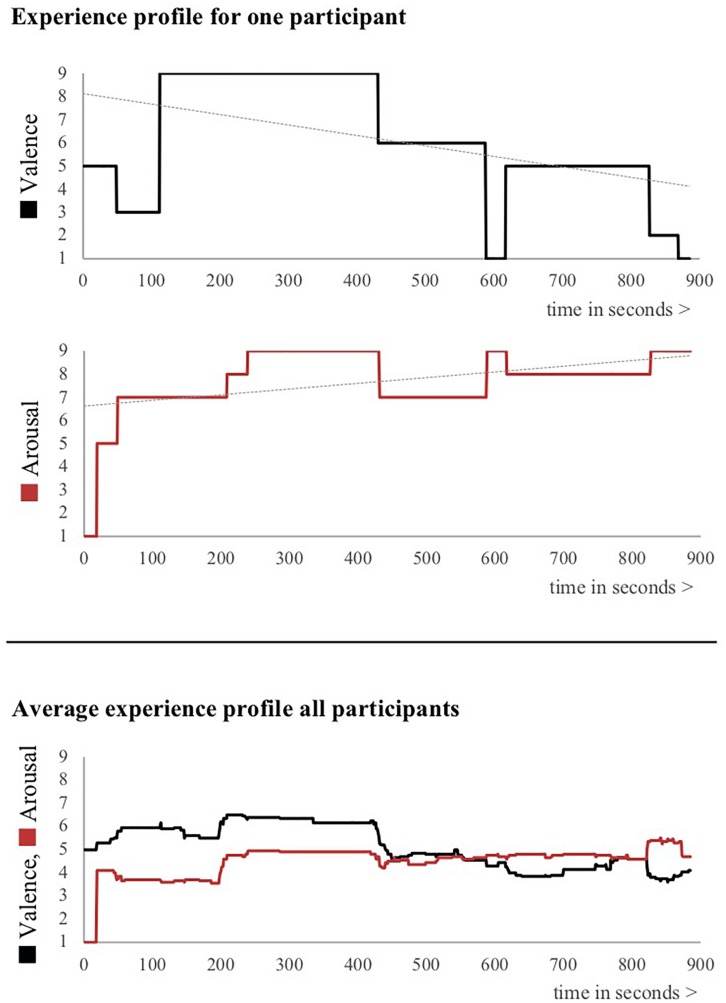
Experience profiles for one participant in terms of valence and arousal (above), as well as the grand average over all participants (below). Note that the individual experience profiles, such as the one presented in the top figure, show higher and lower ratings for both valence and arousal, but that these cancel out in the grand average because of the averaging procedure.

Next, the experimenter asked participants to rate *each segment separately* using the same assessment procedure, with the segments being defined on the basis of conjunction words in the participant’s retellings. After this procedure, the experimenter rewatched the movie together with the participant. Participants were asked to indicate the exact onset and offset times for each of the segments from their retelling, using a time indicator provided on the screen. Combining the individual segments’ onset and offset times with the valence and arousal ratings for each segment, a temporal profile of emotional valence and arousal during the experience was reconstructed (see Figure 2). These profiles served as a representation of the actual, immediate experience.

Approximately 7 days later (*M* = 8.70; *SD* = 3.25), participants were invited back to the lab, to assess their overall evaluations at a second point in time. They were asked to provide overall evaluations of the movie again, using the same scales as mentioned above. Finally, participants were asked to indicate the most memorable moment of the movie and to rate this moment on valence and arousal.

### Data Analysis

From the reconstructed experience, we calculated the following parameters (see Table 2 for an operationalization scheme): *for valence*: peak, trough, end, an average of peak and end [peak-end valence], an average of trough and end [trough-end valence], valence at the most memorable moment [MMM valence], average valence, valence variability and valence slope; *for arousal*: peak, end, an average of peak and end [peak-end arousal], arousal at the most memorable moment [MMM arousal], average arousal, arousal variability and arousal slope. As the method of segmentation did not deliver valence and arousal ratings in terms of single time points, but ratings that were smeared out over a full segment, it was difficult to point out one moment in time from which peak latency and end-after-peak latency could be calculated. Peak latency and end-after-peak latency were therefore not included in the analysis.

**TABLE 2:** Operationalization and descriptive statistics for the different valence and arousal measures.

**Variable**	**Operationalization**	***M* (*SD*)**
***Per-segment valence ratings***		
Positive peak	Most positive valence rating	7.300 (1.043)
Negative peak	Most negative valence rating	2.400 (1.105)
End	Valence rating during final segment	3.775 (1.847)
Peak-end	Average of peak and end valence	5.538 (1.117)
Trough-end	Average of trough and end	3.088 (1.270)
Average	Average of valence ratings across segments	4.938 (0.819)
Variance	Variance of valence ratings across segments	3.328 (2.522)
Slope (valence/second)	Linear trend of valence ratings over segments	−0.003 (0.002)
***Overall valence ratings***		
Immediate overall valence	Scale	5.875 (1.418)
Later overall valence (1 week later)	Scale	5.075 (2.080)
***Per-segment arousal ratings***		
Peak	Most intense arousal rating	6.875 (1.636)
End	Arousal rating during final segment	5.075 (2.165)
Peak-end	Average of peak and end arousal	5.975 (1.768)
Average	Average of arousal ratings across segments	5.032 (1.524)
Variance	Variance of arousal ratings across segments	2.115 (1.429)
Slope (arousal/second)	Linear trend of arousal ratings over segments	0.002 (0.003)
***Overall arousal ratings***		
Immediate overall arousal	Scale	4.350 (2.095)
Later overall arousal (1 week later)	Scale	5.675 (1.716)

Per parameter, an ordinary least squares regression analysis was then performed in order to predict overall experience evaluations. Each parameter served as a predictor variable; the outcome variables were the overall evaluations of valence and arousal as measured directly after having seen the video [*immediate remembered valence* and *immediate remembered arousal*]. Separate regression analyses were used in a similar fashion, to predict overall evaluations of valence and arousal approximately 7 days later [*later remembered valence* and *later remembered arousal*] as outcome variables. To reduce the family-wise error rate, we used a Bonferroni correction in assessing the significance of the different regression models, yielding criteria for significance of α_FW_ = 0.006 for valence predictors and α_FW_ = 0.007 for arousal predictors (based on 9 and 7 ordinary least squares regressions in one family of tests, for valence and arousal, respectively). In order to compare the predictive value of the different predictors, we report, for each regression the significance (*p*-values) of the *F*-statistic and the coefficient of determination (*R*^2^-values).

## Results

### Ratings and Segmentation

On average, participants segmented the movie into 11.03 segments (*SD* = 2.54; *min* = 7; *max* = 18). Across participants, segments had an average duration of 57.25 s (*min* = 1; *max* = 373).

Descriptive statistics for all valence and arousal ratings are presented in Table 2. For the per-segment ratings, valence ranged from 1 to 9 and the average rating of all ratings across segments and participants was very close to the neutral score of 5 (*M* = 4.94; *SD* = 0.82). Per-segment arousal ratings ranged from 1 to 9 and the average across segments and participants was moderately arousing (*M* = 5.04; *SD* = 1.52). Note that although the grand average in Figure 2 shows that experience profiles averaged across participants yield moderate valence and arousal ratings, the experience profiles of individual participants show valence and arousal ratings with substantial deflections from the neutral valence and arousal points. These deflections cancel out in the mean experience profile due to the process of averaging, which underlines the individual nature of experiences. Remembered valence immediately after watching the VR movie was mildly positive (*M* = 5.88; *SD* = 1.42), but approximately 1 week later this dropped back to neutral levels (*M* = 5.08; *SD* = 2.08). The difference was significant (*t*_39_ = 3.035; *p* = 0.004). Somewhat surprisingly, immediately remembered arousal was lower (*M* = 4.35; *SD* = 2.09) than remembered arousal approximately 1 week later (*M* = 5.68; *SD* = 1.72). This difference proved to be significant as well (*t*_39_ = −3.981; *p* < 0.001). As such, immediately remembered valence and arousal and later remembered valence and arousal prove to be different from each other, yet in different directions.

### Analyses on Emotional Valence

Results from the regression analyses for valence are presented in Table 3. The analyses indicate that the effects are of substantial size with *R*^2^-values ranging from 0.23 to 0.46. The results further show that immediate overall valence can be significantly predicted from peak valence, average valence and valence variance. Peak valence was the best predictor, as it accounted for 41.9% of the variance in immediate overall valence, whereas average valence and variance of valence predict only 29.3 and 23.8%, respectively. Trough, end, peak-end, trough-end, MMM and slope were not significant.

**TABLE 3:** Results of linear regression analyses for valence.

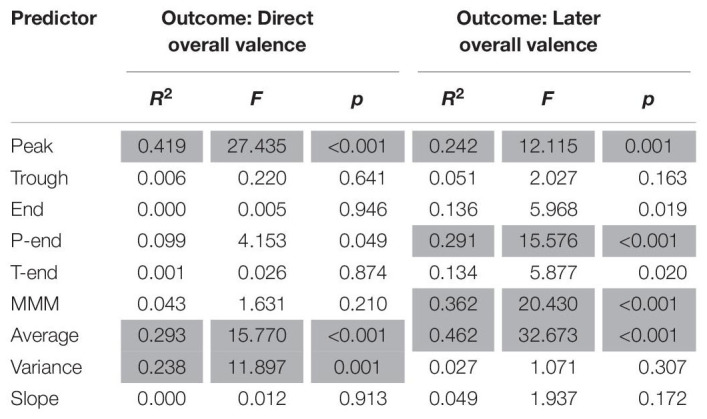

*Due to a Bonferroni correction, α is set at 0.006. Significant predictors are marked in gray.*

For overall valence 1 week later, the results show that this can be significantly predicted from peak valence, peak-end valence, MMM valence and average valence. Average valence was the best predictor, as it accounted for 46.2% of the variance in later overall valence. This is higher than the variance accounted for by MMM valence (36.2%), peak valence (24.2%) and peak-end valence (29.1%). Trough, end, trough-end, variance and slope do not significantly account for any variance in overall valence as measured 1 week later.

We also analyzed whether both direct and remembered valence could be predicted by arousal predictors (peak arousal, end arousal, peak-end arousal, MMM arousal, average arousal, arousal variance and arousal slope). As presented in Table 4, the results show that neither direct remembered valence nor later remembered valance can be predicted from emotional arousal parameters.

**TABLE 4:** Results of linear regression analyses for valence with arousal predictors.

**Predictor**	**Outcome: Direct overall valence**			**Outcome: Later overall valence**		
	***R*^2^**	***F***	***p***	***R*^2^**	***F***	***p***
Peak	0.091	3.821	0.058	0.002	0.069	0.794
End	0.098	4.148	0.049	0.016	0.624	0.434
P-end	0.110	4.679	0.037	0.009	0.361	0.551
MMM	0.003	0.121	0.730	0.000	0.003	0.958
Average	0.079	3.256	0.079	0.001	0.037	0.848
Variance	0.076	3.123	0.085	0.009	0.338	0.564
Slope	0.012	0.448	0.507	0.008	0.289	0.594

*Due to a Bonferroni correction, α is set at 0.007.*

### Analyses on Emotional Arousal

Results from the regression analyses for arousal are presented in Table 5. Again, the analyses indicate that the effects are of substantial size with *R*^2^-values ranging from 0.21 to 0.47. It can be seen that peak arousal, end arousal, peak-end arousal, MMM arousal and average arousal significantly predict immediate overall arousal. With 47.2% of the variance accounted for, average arousal was the best predictor, followed by MMM accounting for 41.0% variance in immediate overall arousal. Peak, end and peak-end only explained 21.6, 24.2, and 26.5%, respectively. Immediate overall arousal was not significantly predicted by variance of arousal and arousal slope.

**TABLE 5:** Results of linear regression analyses for arousal.

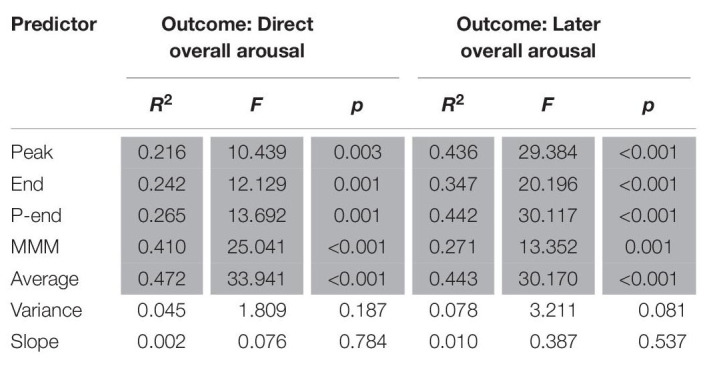

*Due to a Bonferroni correction, α is set at 0.007. Significant predictors are marked in gray.*

With regards to arousal as measured 1 week later, the results also suggest that peak arousal, end arousal, peak-end arousal, MMM arousal and average arousal significantly predict later overall arousal. Again, average arousal is the best predictor, accounting for 44.3% of the variance in later overall arousal. Note, however, that the explained portions of variance for peak arousal (43.6%) and peak-end arousal (44.2%) are fairly close to that of average arousal. End arousal and MMM arousal explain 34.7 and 27.1% of variance in later overall arousal, respectively. Variance of arousal and arousal slope did not significantly predict later overall arousal.

We also analyzed whether both direct and remembered arousal could be predicted by valence predictors (peak valence, trough valence, peak-end valence, trough-end valence, end valence, MMM valence, average valence, valence variance and valence slope). As presented in Table 6, the results show that direct remembered arousal can be significantly predicted from trough valence, trough-end valence and valence variance. Trough valence is the best predictor, accounting for 24% of the variance in direct overall arousal, followed by trough-end valence (21%) and valence variance (18.7%). Later remembered arousal, however, could not be predicted from emotional valence parameters.

**TABLE 6:** Results of linear regression analyses for arousal with valence predictors.

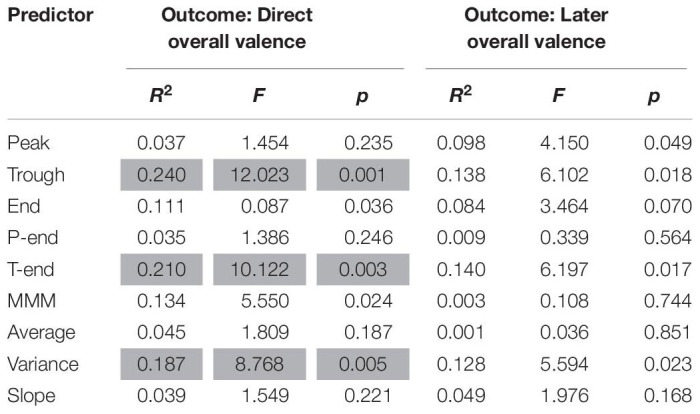

*Due to a Bonferroni correction, α is set at 0.006. Significant predictors are marked in gray.*

## Discussion and Conclusion

This study assessed the robustness of the PE-rule in relating immediate experience to remembered experience. Specifically, we addressed the robustness of the PE-rule 1) for ecologically more valid, heterogeneous experiences and 2) for predicting not only immediately remembered, but also later remembered experience (here 1 week later). In addition, we explored the predictive power of parameters of immediate experience other than peak, end and an average of peak-end valence, namely: peak arousal, end arousal, peak-end arousal, trough valence, trough-end valence, average valence and arousal, valence and arousal at the most memorable moment, valence and arousal variance and valence and arousal slopes. Participants watched a virtual reality movie, after which they were asked to retell what they had just experienced in detail. From their reconstructed experience, the above-listed parameters were then used to predict immediately and later remembered valence and arousal. Results indicate that immediately remembered overall valence and arousal are best predicted by peak emotional valence and by average emotional arousal, while remembered overall valence and arousal after 1 week are best predicted by average emotional valence and average emotional arousal. Thus, our results contrast with the predictions based on the PE-rule.

### Average Is a Better Predictor Than Peaks and Ends

In our study, average affect seems to be the best predictor for remembered overall valence and arousal. Only for immediately remembered overall valence, peak valence proves to be the strongest predictor. In all other cases, average valence and arousal prove to be the best predictor for overall evaluations of an experience. These data suggest that for rich, heterogeneous experiences, the PE-rule is not the best measure for explaining the relationship between experience and memory. This observation is in line with findings from several other studies that evaluated more heterogeneous experiences (Cojuharenco and Ryvkin, 2008; Kemp et al., 2008; Seta et al., 2008; Miron-Shatz, 2009; Schneider et al., 2011). Together, the available evidence converges onto the notion that the PE-rule does not apply for more heterogeneous and ecologically valid experiences, such as the current VR experience, or the experiences that make up everyday life.

As said, however, the majority of extant studies, in which more homogeneous and/or negatively valenced (e.g., undergoing colonoscopy) experiences were studied (e.g., Redelmeier and Kahneman, 1996; Ariely, 1998; Chajut et al., 2014) have provided support for the PE-rule. This begs the question of why the relationship between immediate experience and remembered experience is so markedly different for homogeneous versus richer, heterogeneous experiences. A possible answer may be that more complex experiences are not easily captured by focusing only on two selected moments of the experience (the peak and the end), because there are many more moments that contribute to the overall remembered experience. Another possibility may be that peak and end measures work well when only positive or negative valence is experienced, but that this relationship gets disrupted when both positive and negative emotions are induced during the experience, yielding both peaks and troughs in emotional valence. As our data do not differentiate between these alternatives, this issue may be addressed in future work.

### The PE-Rule Loses Its Predictive Power Over Time

Another finding is that peak valence best predicts immediate overall valence, but not later overall valence (measured 1 week later). We observed that instead of peak valence, average valence is a better predictor for later overall valence. In fact, average valence more strongly predicts later remembered valence than immediately remembered valence (46.2% versus 29.3%, respectively). Moreover, average arousal showed to be the best predictor both for immediate and for later overall arousal. These data suggest that the PE-rule loses its predictive power when time elapses, away from the actual experience. Our data are in line with findings from Geng et al. (2013), although these authors report the PE-rule to lose its predictive power between 3 and 7 weeks after the experience. However, in the study of Geng et al. (2013) experiences consisted of holiday breaks extending over one to several weeks, so the difference in time frame of the experience may have had an influence on the estimated time frame for the PE-rule to hold. Apart from those details, however, the evidence suggests that the predictive power of peaks and ends is relatively short-lived, which limits the value of the PE-rule in many practical situations.

### Emotional Arousal Versus Emotional Valence

This study is the first PE-study to include emotional arousal besides emotional valence. Findings show that arousal was not only different from valence with regards to its significant predictors for overall evaluations (for arousal, the PE-related predictors were all significant, whereas for valence, only peak and peak-end were observed to be significant predictors). In addition, emotional arousal was more persistent in its predictive value over time than emotional valence. Also, in cross-dimensional analyses, it was shown that arousal parameters do not significantly predict immediately and later remembered valence. However, a selective amount of valence parameters do significantly predict immediately remembered arousal (i.e., trough valence, trough-end valence and valence variance). In our data, later remembered arousal cannot not predicted by valence parameters. Exactly how valence and arousal differently affect the memory trace of an experience remains to be studied more systematically, but our data show at the very least that emotional arousal is a factor that should be entered into the equation when studying the relationship between experience and memory.

### Further Considerations

In this study, we invoked a more heterogeneous experience through the use of a movie that evokes a succession of both positive and negative emotions. This, study, however, did not directly compare this heterogeneous experience to the more homogeneous experiences from earlier studies on the PE-rule. A suggestion for further research could be to introduce a factor of homogeneity or heterogeneity as one of the experimental manipulations. Also, we attempted to create an ecologically more valid experience, while still maintaining experimental control through the use of a virtual reality environment. The reason for doing so was to induce an experience type that would lie closer to the experiences in everyday live. Although virtual reality can enhance such ecological validity in an experimental setting (Diemer et al., 2015; Parsons, 2015), virtual reality is still “virtual” and not “real” and as such we only approach ecological validity to some extent. Furthermore, the presentation of a movie is only one of the many experiences that can occur in everyday life, with a particular level of heterogeneity. Generalizing our results to the wide variety of experiences that make up everyday life is therefore a hazardous enterprise and studies that address the issue of ecological validity with a larger variety of experiences would therefore be very welcome.

One substantial limitation in our current work is that we make use of a retrospective method to collect information about ongoing experience. This retrospective approach was adopted to avoid the methodological problems of experience sampling techniques, which have been used in earlier studies (Ariely, 1998; Ariely and Zauberman, 2000). Experience sampling has the disadvantage that the process of measurement interferes with the process to be measured, i.e., that experience sampling at regular time intervals disrupts, or at least transforms the experience. However, a disadvantage of the currently used retrospective approach is that although the time between the actual experience and our surveying of the participants was kept to a strict minimum, our measure of ongoing experience was still reconstructed from retrospective data. This has several implications. A first implication is that, strictly speaking, the retrospective approach measures remembered experience instead of actual real-time lived experience. As the PE-rule is meant to describe relationships between experience and memory, it is not ideal to reconstruct ongoing experience measures from memory itself. In our view, though, the approach in this paper is the closest possible way to measuring self-reported, lived experience without disrupting the experience itself, as is the case with traditional experience sampling methods. Nonetheless, it is known that experience and memory are quite different in nature (see e.g., Kahneman and Riis, 2005), and hence may predict overall evaluations differently. Further research to compare retrospective approaches with real-time approaches to measure immediate experience is therefore much recommended. A second implication is that rather than extracting predictors from an ongoing, real-time measure of lived experience, predictors are now based on an experience that is segmented into episodes. The resulting predictors may be different from those that are based on real-time measures, because of the aggregation within segments of an episode. For example, it may be that actual valence varies within such a segment between positive and negative values, which is obscured when participants are asked to give a single rating for the whole segment. Another drawback of our segmentation procedure is that it required additional processing from the participants, which may affect the effect of time (immediate overall ratings versus 1 week-later overall ratings).

With the predictors currently used, the maximum proportion of variance in remembered overall evaluations lies between 40 and 50%. Although this is rather substantial, this leaves us with around 60 to 50% of unexplained variance. This raises the question of whether there are other experience proxies that might explain more variance, in addition to the currently used predictors. The vast majority of PE-studies makes use of emotional responses measured through self-report (either verbally, in print or through a response device). Beyond the field of self-report is a whole field of psychophysiological measurement, in which physiological measures such as heart rate, skin conductance or facial electromyography are thought to yield reliable indices of affective engagement (see e.g., Mauss and Robinson, 2009; Kreibig, 2010; Bastiaansen et al., 2019), without being as disruptive to the experience as using self-report. Recently, wearable measurement devices have been introduced that enable researchers to measure changes in physiology over time in truly ecologically valid settings, such as city trips and walks (Kim and Fesenmaier, 2014; Shoval et al., 2017). Further research could explore whether using such psychophysiological indices may further improve our understanding of the relationship between affect, experience and memory.

## Conclusion

In conclusion, we show that the predictive value of the PE-rule, which describes a possible relationship between experience and memory, is limited for experiences with a rich and heterogeneous nature that are closer to everyday life experiences compared to the experiences that have been used in previous studies. Furthermore, the PE-rule loses its power to predict the evaluation of an experience with time. Our results suggest that average experience may be a better predictor to describe the relationship between the emotions in our experiences and how they are remembered. Additionally, we show that measuring emotional arousal in addition to emotional valence may improve prediction of the memory of an experience. As such, it may be beneficial to incorporate emotional arousal in further studies on emotional experiences and how they will be remembered.

## Data Availability

The datasets generated for this study are available on request to the corresponding authors.

## Ethics Statement

This study was carried out in accordance with the recommendations of EC-2016.48 of the Ethics Review Board of Tilburg University with written informed consent from all subjects. All subjects gave written informed consent in accordance with the Declaration of Helsinki. The protocol was approved by the Ethics Review Board of Tilburg University.

## Author Contributions

All authors contributed to the conception and design of the study, manuscript revision, and read and approved the submitted version. MvG and MD designed the stimulus for the study. WS, MB, and JG organized the database and performed the statistical analysis. WS wrote the first draft of the manuscript. OM, MB, and JG wrote the sections of the manuscript.

## Conflict of Interest Statement

The authors declare that the research was conducted in the absence of any commercial or financial relationships that could be construed as a potential conflict of interest.
